# Prepartum supplementation of conjugated linoleic acids (CLA) increased milk energy output and decreased serum fatty acids and β-hydroxybutyrate in early lactation dairy cows

**DOI:** 10.1371/journal.pone.0197733

**Published:** 2018-05-17

**Authors:** Rafael Caputo Oliveira, Ryan S. Pralle, Lucas C. de Resende, Carlos Henrique P. C. Nova, Valentina Caprarulo, Joshua A. Jendza, Arnulf Troescher, Heather M. White

**Affiliations:** 1 Department of Dairy Science, University of Wisconsin-Madison, Madison, Wisconsin, United States of America; 2 Department of Medicine Veterinary, Federal University of Lavras, Lavras, Minas Gerais, Brazil; 3 Department of Animal Science, State University of Northern Rio de Janeiro, Campos dos Goytacazes, Rio de Janeiro, Brazil; 4 Department of Health, Animal Science and Food Safety, University of Milan, Milan, Italy; 5 BASF, Florham Park, New Jersey, United States of America; 6 BASF, Lampertheim, Hesse, Germany; University of Illinois, UNITED STATES

## Abstract

Prepartum supplementation with conjugated linoleic acid (CLA) may influence lipolysis and hyperketonemia in dairy cows. The objective of this study was to examine the effect of prepartum CLA supplementation on lactation performance and serum fatty acids (FA) and β-hydroxybutyrate (BHB) in early lactation dairy cows, and secondarily on reproductive performance. Multiparous cows were enrolled in the study at 18 days prior to expected calving date, and randomly assigned 100 g/day of Lutrell Pure (BASF, Ludwigshafen, Germany; 75% FA), providing 10 g/day of each CLA isomer (*trans*-10 *cis*-12 and *cis*-9 *trans*-11 CLA) or equivalent amount of rumen inert fatty acids as control (78 g/day of Energy Booster 100; Milk Specialties Global, Eden Prairie, MN). Treatments were top dressed daily to individual cows from enrollment to calving and all cows were offered the same ration. Blood samples were collected on the first day of supplementation, 10 days prepartum, and 1, 7, 14, and 30 days postpartum. Hyperketonemia was defined as serum BHB ≥ 1.2 mM. Milk yield was recorded daily until 60 days postpartum and averaged weekly. Milk samples were obtained weekly for component analysis. Prepartum CLA supplementation tended to increase serum concentration of cis-9, trans-11 CLA and increased trans-10, cis-12 CLA prepartum. Cows supplemented with CLA had increased milk protein yield and tended to have increased milk fat yield and milk yield, which together resulted in greater energy content of milk. Cows supplemented with CLA had lower serum FA on day 1 and 7 postpartum and overall lower serum BHB postpartum, which resulted in decreased prevalence of hyperketonemia on day 14 postpartum. There were no differences in body condition score change, other health disorders, or reproductive outcomes by treatment. Together, these findings indicate that prepartum CLA supplementation may be a plausible strategy to positively influence postpartum performance.

## Introduction

Physiological changes during pregnancy in dairy cows, coupled with negative energy balance, results in mobilization of body reserves during the transition from gestation to lactation. Excessive mobilization of fatty acids (FA) can surpass hepatic complete oxidative capacity leading to an excessive production of ketone bodies [1, 2]. Production of ketone bodies beyond oxidative capacities of tissues results in hyperketonemia (HYK), which is associated with negative impacts on production, fertility, and health in dairy cows [3, 4].

Supplementation of conjugated linoleic acid (CLA) in the early postpartum period is a possible strategy to decrease negative energy balance and improve postpartum metabolic health by reducing milk fat secretion [5–7]. This scenario is undesirable by many dairy producers who sell milk in component compensated markets. Supplementation of CLA pre- and postpartum also reduced plasma FA during the early postpartum period compared to cows only supplemented after calving with the same doses of CLA or no supplementation [8]. The authors attributed the lower plasma FA in cows supplemented pre-and postpartum to a lower BCS loss observed, suggesting that these effects required either prepartum or both pre- and postpartum CLA supplementation. Supplementing CLA exclusively prepartum would allow the investigation of the effect of CLA on reducing blood FA in the peripartum period in dairy cows. Furthermore, the use of this strategy would not likely decrease milk fat synthesis since the half-life of plasma trans-10, cis-12 CLA and the half-life of enzymes responsible for milk fat synthesis subject to regulation by this CLA isomer are less than 3 days [9–11].

We hypothesize that prepartum CLA supplementation in dairy cows could be a strategy to decrease fat mobilization around calving, decrease postpartum prevalence of HYK, and improve lactation performance without reducing milk fat synthesis. Thus, the objective of this study was to examine the effects of CLA supplemented exclusively in the prepartum transition period on lactation performance and postpartum serum FA and β-hydroxybutyrate (BHB) in dairy cows. A secondary objective was to examine the effect of prepartum CLA supplementation on subsequent reproductive performance.

## Materials and methods

All animal use and handling protocols were approved by the University of Wisconsin-Madison College of Agricultural and Life Sciences Animal Care and Use Committee. The study was conducted on a privately owned dairy farm in Wisconsin, USA. All cows were housed in the same cross-ventilated free stall barn for the entire trial.

Multiparous cows were moved from the far off dry cow pen to the prepartum transition period pen once per week between -25 and -17 days from the predicted calving date. Cows were blocked by expected calving date and randomly assigned to a treatment once moved. The process was repeated weekly until all cows were enrolled (n = 314). Treatments were 100 g/day of Lutrell Pure (BASF, Ludwigshafen, Germany; 75% FA), providing 10 g/day of each CLA isomer (*trans*-10 *cis*-12 and *cis*-9 *trans*-11 CLA) or equivalent amount of rumen inert FA as control (78 g/day of Energy Booster 100; Milk Specialties Global, Eden Prairie, MN; 96.1% FA: 46.2% C18:0, 28.0% C16:0, and 8.3% C18:1 cis-9). The CLA methyl esters in Lutrell Pure are lipid-encapsulated to be insoluble and resistant to ruminal biohydrogenation processes. Supplementing the same amount of fatty acids to both treatments was a strategy to avoid the potential confounding effect of energy intake. Daily supplementation began at the start of the prepartum transition period until calving, immediately prior to the once a day total mixed ration feeding (1500 hours). Before the treatments were provided, all cows in the pen were locked into headlocks which allowed for individual supplementation of each cow. Both treatments were mixed with 200 g/cow/day of corn gluten feed and provided on the clean feed bunk surface to individual cows. The treatment intakes were observed visually by four researchers until complete consumption and recorded daily. After calving, all cows received the same formulated diet until the end of the study. The period from the beginning of treatment allocations until the day the last cow completed 150 days postpartum lasted from October 27, 2015 to June 26, 2016. Parturitions occurred from November 7, 2015 to January 28, 2016.

The composition of the diets were determined by the private dairy manager and nutrition consultant, without the interference of the research team. Total mixed ration samples were collected each week. Samples were placed in a 55 °C forced-air oven for 48 hours, ground to pass a 1-mm screen (Wiley Mill; Arthur H. Thomas, Philadelphia, PA, USA), and composited by month. Chemical composition was evaluated at a commercial laboratory (Dairyland Labs, Arcadia, WI). The basal diets without treatments for the pre- and postpartum transition period are presented in Table 1. Feed fatty acid content and composition was determined by gas chromatography using C19:0 as an internal standard as described previously [12]. Fatty acids were 3.8% ± 0.15 of the prepartum of diet DM (excluding treatments). The fatty acid profile (as a percent of total fatty acids) of the prepartum ration was C14:0 (1.0% ± 0.22), C16:0 (20.8% ± 0.84), C16:1 cis-9 (1.2% ± 0.11), C18:0 (4.2 ± 0.84), C18:1 cis-9 (11.8% ± 0.59), C18:2 cis-6 and 9 (27.6% ± 2.33), and C18:3 cis-3, 6, and 9 (21.0% ± 2.63). Conjugated linoleic acid isomers were not detected in feed samples.

**Table 1:** Ingredient and chemical composition of basal diets fed during the pre- and postpartum period^a^.

Item	Prepartum	Postpartum
Ingredient, % of dry matter		
Corn silage	22.1	34.6
Alfalfa silage	-	24.5
Ryegrass silage	65.6	-
Whey	-	4.0
Postpartum concentrate^b^	-	11.6
High moisture corn	-	13.2
Soybean meal	-	4.0
Tallow	-	0.6
Corn gluten feed	-	7.4
Prepartum concentrate^c^	12.2	-
Chemical composition^d^, mean ± standard deviation		
Dry matter, % as fed	46.2 ± 0.63	44.4 ± 1.31
Crude protein, % of dry matter	16.5 ± 1.06	17.2 ± 1.24
Neutral detergent fiber, % of dry matter	37.7 ± 3.70	28.6 ± 3.45
Starch, % of dry matter	10.2 ± 1.79	23.7 ± 1.44
Sugar^e^, % of dry matter	6.6 ± 0.56	4.6 ± 0.74
Fat^f^, % of dry matter	5.5 ± 0.51	4.7 ± 0.35
Ash, % of dry matter	11.6 ± 0.67	6.9 ± 0.50
Non-fiber carbohydrates^g^, % of dry matter	28.7 ± 3.02	42.5 ± 2.98

^a^ Prepartum (between -25 to -17 days of the predicted calving date until calving). Postpartum (calving until 150 days postpartum).

^b^ SoyPlus (34.2%; West Central Cooperative, Ralston, IA), ground corn (33.2%), calcium carbonate (6.3%), SQ810 (6.3%; Arm and Hammer, Princeton, NJ), soybean meal (5.3%), Megalac-R (4.2%; Arm and Hammer, Princeton, NJ), urea (4.2%), sodium chloride (2.1%), magnesium oxide (2.1%), Celmanax (0.6%; Arm and Hammer, Princeton, NJ), Dairy Balancer 11 (0.5%; Quality Liquid Feeds, Dodgeville, WI), Alimet (0.47%; Novus International, St. Charles, MO), Mepron-M85 (0.47%; Evonik Nutrition and Care GmbH, Hanau-Wolfgang, Germany), AjiPro-L (0.42%; Ajinomoto Co. Inc., Tokyo, Japan), Feed-Bond (0.32%; ACG Products Ltd., Brookfield, WI), Availa-ZN 120 (0.11%; Zinpro Corporation, Eden Prairie, MN), Rovimix Biotin (0.06%; DSM Nutritional Products, Belvidere, NJ), Rumensin-90 (0.05%; Elanco Animal Health, Greenfield, IN).

^c^ Ground corn (39.7%), Soyplus (20.0%; West Central Cooperative, Ralston, IA), calcium chloride (14.7%), magnesium sulfate (9.3%), Megalac-R (6.7%; Arm and Hammer, Princeton, NJ), Vicomb (2.7%; Vetagro Inc., Chicago, IL), magnesium oxide (1.3%), sodium chloride (1.3%), Dairy Balancer 11 (1.3%; Quality Liquid Feeds, Dodgeville, WI), Celmanax (0.8%; Arm and Hammer, Princeton, NJ), Feed-bond (0.8%; ACG Products Ltd., Brookfield, WI), Mepron-M85 (0.4%; Evonik Nutrition and Care GmbH, Hanau-Wolfgang, Germany), Alimet (0.4%; Novus International, St. Charles, MO), Rovimix E50 (0.2%; DSM Nutritional Products, Belvidere, NJ), Rovimix Biotin (0.16%; DSM Nutritional Products, Belvidere, NJ), Rumensin-90 (0.08%; Elanco Animal Health, Greenfield, IN), Availa-ZN (0.05%; Zinpro Corporation, Eden Prairie, MN).

^d^ Nutrient composition from 3 and 5 composite samples for pre- and postpartum diets without treatments, respectively.

^e^ Water soluble carbohydrates.

^f^ Ether extract.

^g^ Non-fiber carbohydrates, % = 100—(Neutral detergent fiber, % + crude protein, % + fat, % + ash, %).

Cows were milked three times per day starting at 0530, 1330, and 2130 hours. Milk yield of each cow was recorded daily from 1 to 60 days postpartum and averaged weekly. Individual milk samples were obtained weekly from a midday milking via a composite sampler for milk composition and somatic cells count (SCC) analysis and weekly component yields were estimated as described previously [13]. Milk samples were preserved in 2-bromo-2-nitropropane-1,3-diol (Advanced Instruments, Inc., Norwood, MA, USA), and the milk composition of fat, true protein and lactose was determined by Fourier transform infrared spectrometry using the FOSS MilkoScan FT6000 (FOSS Analytical, Eden Prairie, MN). Analysis of milk SCC was by Fossomatic FC (FOSS Analytical, Eden Prairie, MN). Energy content of milk was determined using the following equation as described by [14]: [(0.0929 x % milk fat) + (0.0547 x % milk true protein/0.93) + (0.0395 x % milk lactose)] x milk yield.

Body condition scores (BCS) were measured at the first day of supplementation (day 18) prior to the expected calving date, and on day 1 and 30 postpartum by two trained individuals using a five-point scale [15]. Both scores were averaged within a timepoint for each cow. Health disorders from calving until 30 days postpartum were defined by the herd veterinarian, and were subsequently retrieved from DairyComp 305 software (Valley Agricultural Software, Tulare, CA).

Blood samples from the coccygeal vessels were obtained from all cows within three randomly selected blocks of cows, totaling a subset of 35 cows per treatment for BHB and FA analysis. Blood samples were collected into tubes without additives (BD Vacutainer, Franklin Lakes, NJ) on the first day of supplementation (18 days prepartum) and on day 10 prior to the expected calving date before feeding time. Blood samples were also collected on days 1, 7, 14, and 30 postpartum. Given the size of pen, cows returned from the parlor over the course of 2 hours and blood samples were collected from individual cows as they returned from milking to fit management schedules. Samples were kept on ice in a cooler until centrifugation. Serum was separated from blood after centrifuging at 2500 x g (g is the gravitational acceleration) for 15 minutes. Serum from each cow and timepoint were aliquoted into tubes and stored at -20° C. Analysis for serum FA concentration was performed using the Wako NEFA-HR(2) Microtiter Procedure kit (Wako Diagnostics, Richmond, VA) and serum BHB concentration was analyzed using the Stanbio BHB LiquiColor kit (Procedure number 2440–058, Stanbio Laboratory, Boerne, TX). The threshold for defining HYK was serum BHB ≥ 1.2 mM. Serum collected at 10 days prior to expected calving date was also analyzed for FA composition to quantify cis-9, trans-11 and trans-10, cis-12 isomers of CLA. Briefly, serum lipids were extracted and FA methylated using 1% methanolic sulfuric acid as described previously [16]. The FA methyl esters of CLA were quantified by gas liquid chromatography as described previously [10].

All cows were synchronized using Double-Ovsynch protocol as previously described [17]. Synchronization and fixed time artificial insemination (FTAI) were performed by a trained farm employee, without the interference of the research team. Services were recorded until 150 days postpartum. Pregnancy diagnosis by transrectal ultrasonography was performed by detecting a viable embryo on day 32 after FTAI. All cows diagnosed pregnant on day 32 were reexamined on day 46 after FTAI. Pregnancy loss was calculated as the difference between cows not pregnant on day 46, but previously diagnosed pregnant on day 32, divided by total number of pregnant cows at day 32.

### Statistical analysis

Continuous variables were analyzed with the MIXED procedure of SAS 9.4 (SAS Institute Inc., Cary, NC). Models for repeated measures contained treatment, time, and interaction of treatment x time as fixed effects and random effects of block and cow(block x treatment). The best covariance structure defined by the Schwarz’s Bayesian Criterion was first order autoregressive. Body condition score changes and serum concentration of the cis-9, trans-11 CLA and trans-10, cis-12 CLA isomers were analyzed separately in a model containing treatment as fixed effect and block as random effect.

Categorical data was analyzed by logistic regression (GLIMMIX, SAS 9.4; SAS Institute Inc., Cary, NC) fitting a binary distribution response. Models for most variables contained treatment as fixed effect and block as random effect. For overall pregnancy per FTAI and overall pregnancy loss, models contained fixed effects of treatment, service, and the interaction of treatment x service and random effects of block and cow(block x treatment).

Rate of pregnancy by day postpartum was analyzed with survival analysis using Cox’s proportional hazards regression model (PROC PHREG, SAS 9.4; SAS Institute Inc., Cary, NC). Model contained treatment as fixed effect and block as random effect. Kaplan-Meier plots with survival curves, mean and median days for calving to conception interval were generated using LIFETEST procedure of SAS 9.4 (SAS Institute Inc., Cary, NC). Comparison of the survival curves between treatments was performed by rank tests for homogeneity: log-rank and Wilcoxon test.

Treatment effects were declared significantly different when *P* ≤ 0.05 and trends toward significance when 0.05 < *P* ≤ 0.10. When *P-value* for interactions of treatment x time was ≤ 0.10, the SLICE option was used to compare treatment differences within individual timepoint. Days of supplementation or physiological state are listed as means ± standard deviation. Treatment effects are listed as least square means ± standard error of the mean (SEM).

## Results

Of the 314 cows enrolled, 284 cows were retained in the experiment for data analysis (control n = 141, CLA n = 143). Cows which refused to consume the treatments during the first five days of supplementation (control n = 2, CLA n = 4) or experienced prepartum health disorders (control n = 9, CLA n = 15) were removed from the study. The number of lactations were 3.1 ± 1.13 and 2.9 ± 0.98 and days of prepartum supplement consumption were 15.6 ± 4.65 and 15.6 ± 4.77 days (mean ± standard deviation) for control and CLA treatments, respectively.

There was no interaction between treatment and time (*P* ≥ 0.22; Table 2) for any milk component percentage and component yield or milk yield. Component yields and percentage, and milk yield increased (*P* < 0.01) as lactation progressed. Cows supplemented with CLA tended to have greater (*P* = 0.09) milk yield. Moreover, cows supplemented with CLA had greater (*P* = 0.01) milk protein yield and percentage, and tended to have greater (*P* = 0.07) milk fat yield, resulting in an increased (*P* = 0.03) milk energy output. Fat and lactose percentage, lactose yield, and Log_10_SCC were not different (*P* ≥ 0.12) between treatments.

**Table 2:** Effect of prepartum control or conjugated linoleic acids (CLA) supplementation on postpartum milk yield, milk component yield and milk composition for dairy cows.

	Treatments^a^			*P-value*		
Item^b^	Control	CLA	SEM	Trt^c^	Time	Trt x time^d^
Milk yield, kg/d	46.6	47.6	0.45	0.09	<0.01	0.67
Milk component yield						
Protein, kg/d	1.38	1.43	0.015	0.01	<0.01	0.69
Fat, kg/d	1.94	2.00	0.026	0.07	<0.01	0.33
Lactose, kg/d	2.28	2.33	0.022	0.12	<0.01	0.71
Milk energy^e^, Mcal/d	35.35	36.36	0.378	0.03	<0.01	0.51
Milk composition						
Protein, %	2.98	3.03	0.017	0.01	<0.01	0.75
Fat, %	4.23	4.25	0.039	0.63	<0.01	0.51
Lactose, %	4.82	4.84	0.012	0.29	<0.01	0.22
SCC^f^, Log_10_ cells x 1000/mL	1.70	1.68	0.047	0.64	<0.01	0.71

^a^ Treatments: Control (n = 141) cows supplemented with 78 g of Energy Booster 100 (Milk Specialties Global, Eden Prairie, MN) and CLA (n = 143) cows supplemented with 100 g/day of Lutrell Pure (BASF, Ludwigshafen, Germany). Supplementation was by individual topdress of product mixed with 200 g corn gluten feed for 16 days prepartum.

^b^ Data from calving to 60 days postpartum, averaged weekly.

^c^ Treatment.

^d^ Interaction treatment x time.

^e^ Energy content of milk was determined using the following equation as described by [14]: [(0.0929 x % milk fat) + (0.0547 x % milk true protein/0.93) + (0.0395 x % milk lactose)] x milk yield.

^f^ Somatic cells count.

For the subset of cows for blood metabolite analysis, six cows experienced prepartum health disorders (control n = 1, CLA n = 5) and were removed. For the 34 and 30 remained cows on control and CLA treatments, respectively, days of prepartum supplement consumption were 17.6 ± 2.99 days and 17.9 ± 2.49 days for control and CLA treatments, respectively. Prepartum CLA supplementation tended to increase (*P* = 0.07) serum concentration of the cis-9, trans-11 CLA isomer (0.22 vs. 0.24% of serum FA) and increased (*P* < 0.01) the trans-10, cis-12 CLA isomer (0.006 vs. 0.015% of serum FA) at 10 days prepartum.

**Fig 1:**
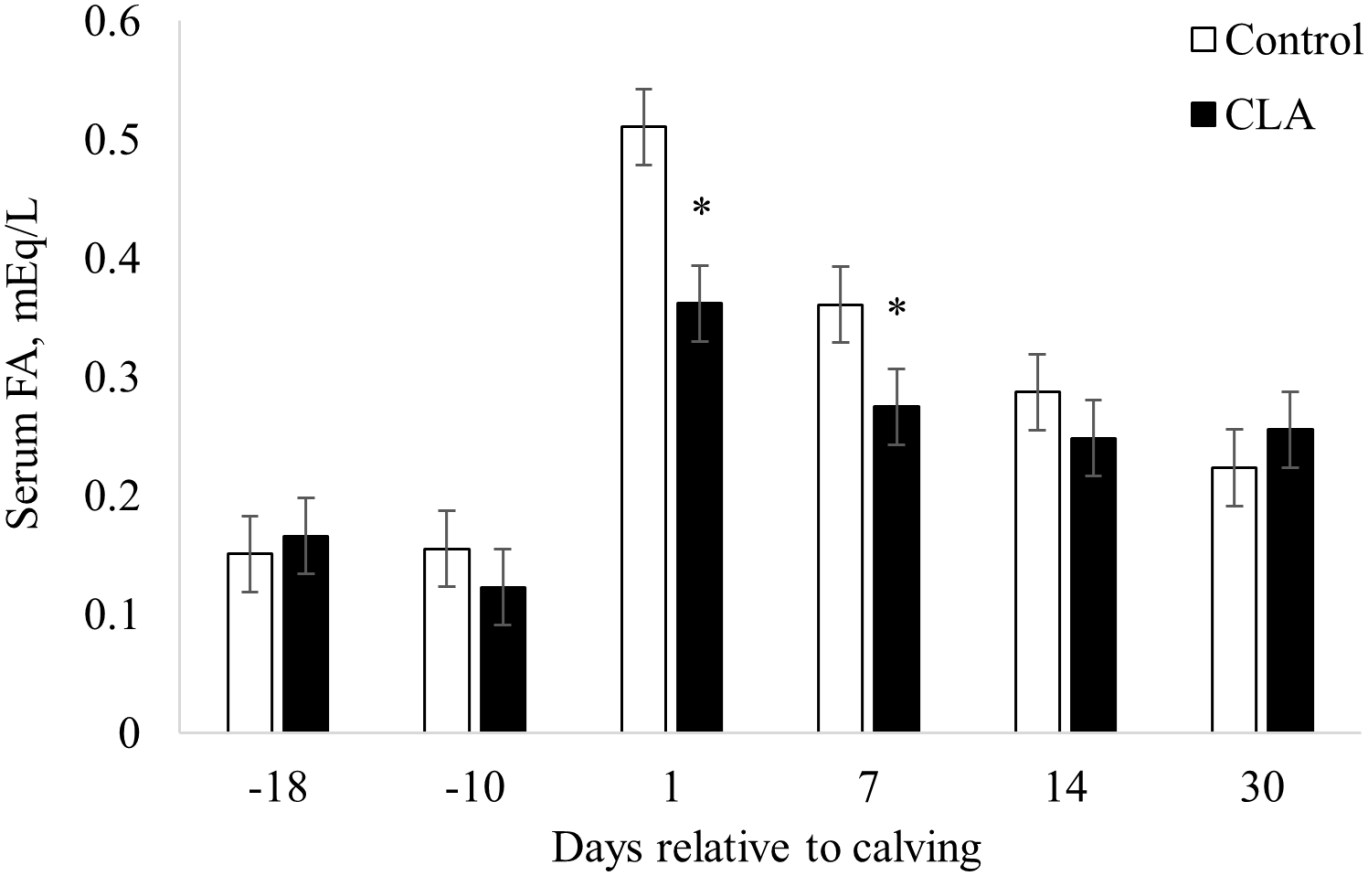
Effect of prepartum control or conjugated linoleic acids (CLA) supplementation on serum fatty acids (FA) at first day of supplementation and 10 days prepartum, and at 1, 7, 14, and 30 days postpartum in dairy cows. Treatments: Control (open bar, n = 34) cows supplemented with 78 g of Energy Booster 100 (Milk Specialties Global, Eden Prairie, MN) and CLA (closed bar, n = 30) cows supplemented with 100 g/day of Lutrell Pure (BASF, Ludwigshafen, Germany). Supplementation was by individual topdress of product mixed with 200 g corn gluten feed for 16 days prepartum. * *P* ≤ 0.05 for treatment differences within individual timepoint. Overall treatment effect *P* = 0.06. Interaction treatment x time *P* = 0.02.

**Fig 2:**
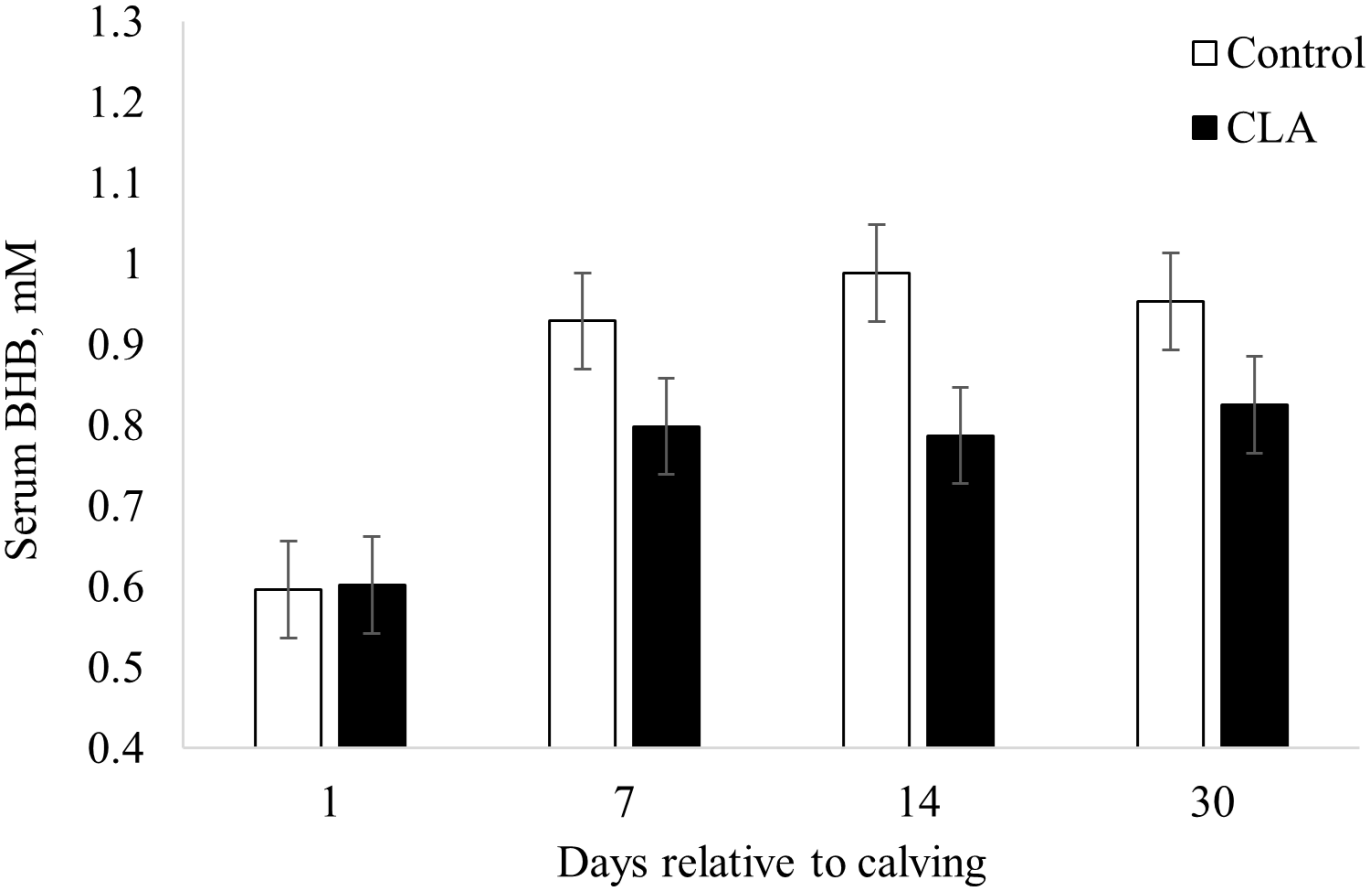
Effect of prepartum control or conjugated linoleic acids (CLA) supplementation on serum β-hydroxybutyrate (BHB) at 1, 7, 14, and 30 days postpartum in dairy cows. Treatments: Control (open bar, n = 34) cows supplemented with 78 g of Energy Booster 100 (Milk Specialties Global, Eden Prairie, MN) and CLA (closed bar, n = 30) cows supplemented with 100 g/day of Lutrell Pure (BASF, Ludwigshafen, Germany). Supplementation was by individual topdress of product mixed with 200 g corn gluten feed for 16 days prepartum. Overall treatment effect *P* = 0.03. Interaction treatment x time *P* = 0.22.

Overall, cows lost (*P* < 0.01) 0.22 BCS points from the day of enrollment to 30 days postpartum (Table 3). Treatment did not affect (*P* ≥ 0.44) BCS or BCS change. Although there was no difference (*P* ≥ 0.47) of prepartum serum FA between treatments, CLA supplemented cows had 31% and 24% lower (*P* ≤ 0.04) serum FA on days 1 and 7 postpartum, respectively (Fig 1). Cows supplemented with CLA also had 13.1% lower (*P* = 0.03) overall postpartum serum BHB (Fig 2), which resulted in a decreased (*P* = 0.05) prevalence of HYK on day 14 postpartum (23.5 vs. 3.3%) in cows supplemented with CLA compared to control cows (Table 4). Prevalence of HYK was not different (*P* ≥ 0.97) on days 1 (2.9 vs. 0%), 7 (8.8 vs. 0%), and 30 (5.9 vs 0%) postpartum between control and CLA supplemented cows, respectively (Table 4).

**Table 3:** Effect of prepartum control or conjugated linoleic acids (CLA) supplementation on body condition score (BCS) and BCS change for dairy cows.

	Treatments^a^			*P-value*		
	Control	CLA	SEM	Trt^b^	Time	Trt x time^c^
BCS^d^	3.03	3.04	0.013	0.44	<0.01	0.65
BCS Change^e^						
-18 to +1	-0.01	-0.01	0.0160	0.81	-	-
+1 to +30	-0.19	-0.19	0.0325	0.96	-	-
-18 to +30	-0.23	-0.21	0.0282	0.51	-	-

^a^ Treatments: Control (n = 141) cows supplemented with 78 g of Energy Booster 100 (Milk Specialties Global, Eden Prairie, MN) and CLA (n = 143) cows supplemented with 100 g/day of Lutrell Pure (BASF, Ludwigshafen, Germany). Supplementation was by individual topdress of product mixed with 200 g corn gluten feed for 16 days prepartum.

^b^ Treatment.

^c^ Interaction treatment x time.

^d^ Data from days 1 and 30 postpartum with day 18 prepartum as a covariate.

^e^ Days relative to calving.

**Table 4:** Effect of prepartum control or conjugated linoleic acids (CLA) supplementation on proportion of health disorders for dairy cows from calving until 30 days postpartum.

	Treatments^a^			
Health disorders, % (n_c_/n_t_)^b^	Control	CLA	SEM	*P-value*
Hyperketonemia				
Day 1 postpartum	2.9 (1/34)	0 (0/30)	3.07	0.98
Day 7 postpartum	8.8 (3/34)	0 (0/30)	4.86	0.97
Day 14 postpartum	23.5 (8/34)	3.3 (1/30)	5.33	0.05
Day 30 postpartum	5.9 (2/34)	0 (0/30)	4.03	0.98
Milk fever	1.2 (2/141)	0.6 (1/143)	0.80	0.59
Retained placenta	4.9 (7/141)	2.1 (3/143)	1.52	0.20
Displaced abomasum	0.7 (1/141)	0.6 (1/143)	0.70	0.99
Mastitis^c^	31.8 (45/141)	31.7 (46/143)	4.66	0.98
Lameness	5.6 (8/141)	4.8 (7/143)	1.94	0.75
Death	1.4 (2/141)	0.7 (1/143)	0.84	0.56

^a^ Treatments: Control (n = 141) cows supplemented with 78 g of Energy Booster 100 (Milk Specialties Global, Eden Prairie, MN) and CLA (n = 143) cows supplemented with 100 g/day of Lutrell Pure (BASF, Ludwigshafen, Germany). Supplementation was by individual topdress of product mixed with 200 g corn gluten feed for 16 days prepartum.

^b^ Number of new cases (n_c_) divided by total number of cows (n_t_).

^c^ Milk samples from cows with somatic cells count greater than 200 cells x 10^3^/mL [18] or diagnosed with clinical mastitis.

**Fig 3:**
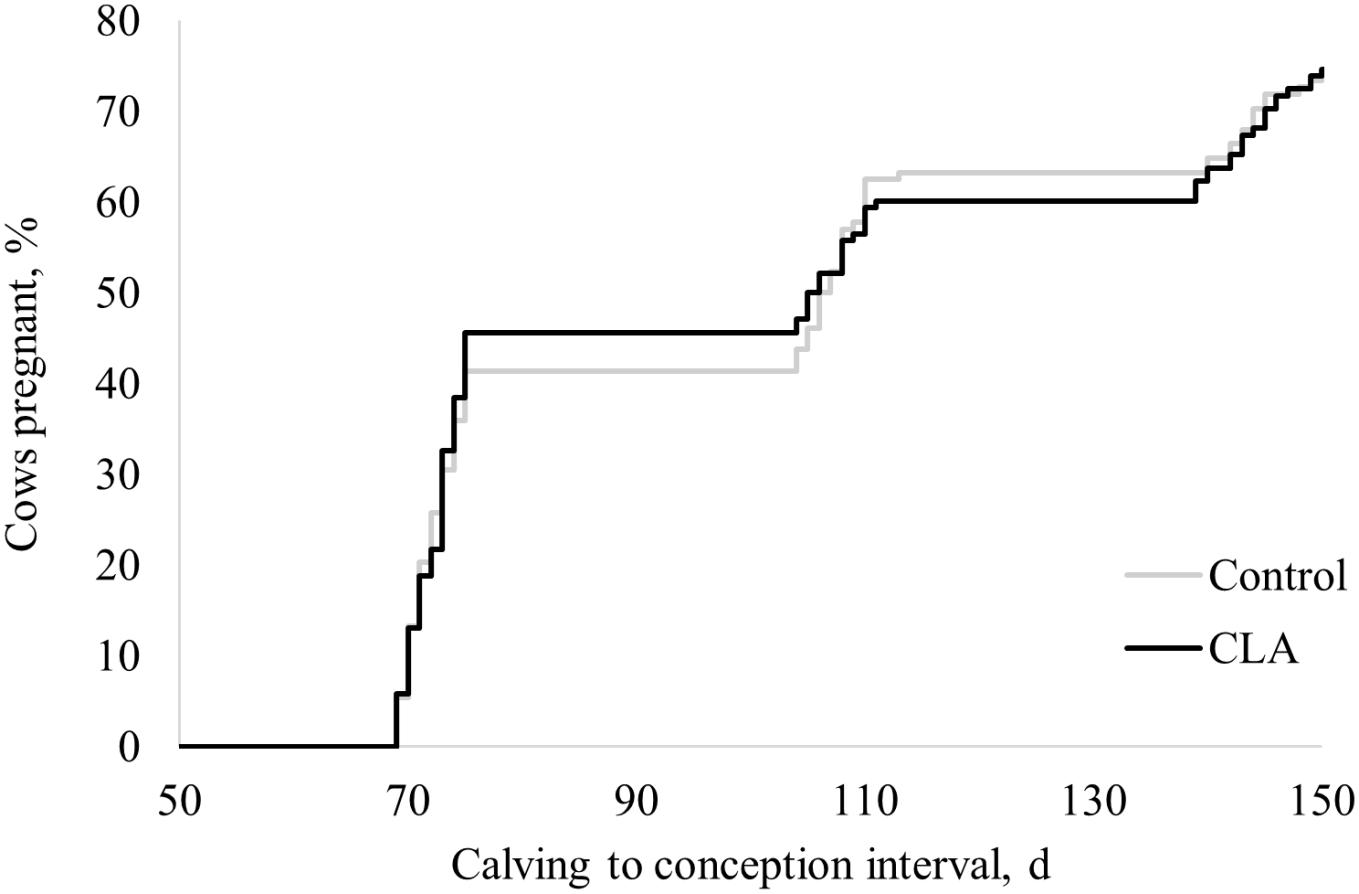
Effect of prepartum control or conjugated linoleic acids (CLA) supplementation on calving to conception interval in dairy cows. Treatments: Control (gray line, n = 128) cows supplemented with 78 g of Energy Booster 100 (Milk Specialties Global, Eden Prairie, MN) and CLA (black line, n = 138) cows supplemented with 100 g/day of Lutrell Pure (BASF, Ludwigshafen, Germany). Supplementation was by individual topdress of product mixed with 200 g corn gluten feed for 16 days prepartum. Hazard ratio (95% confidence interval) and Hazard ratio *P*-value were 0.99 (0.75 to 1.31) and *P* = 0.95. Mean ± standard error were 107.7 ± 3.00 for control and 107.4 ± 2.99 for CLA treatments. Median (95% confidence interval) were 106.5 days (75 to 110 days) for control and 105.5 days (75 to 110 days) for CLA treatments. LogRank *P* = 0.97, and Wilcoxon *P* = 0.96.

There were no differences (*P* ≥ 0.20) between treatments in the proportion of postpartum disorders recorded from calving until 30 days postpartum: milk fever, retained placenta, displaced abomasum, mastitis, lameness, and death (Table 4). Only cows that received FTAI and remained in the experiment until 150 days postpartum (control n = 128, CLA n = 138) were included in the reproductive analysis. For the subset of cows examined for reproductive performance, days of prepartum supplement consumption were 15.7 ± 4.77 days and 15.6 ± 4.62 days. Prepartum CLA supplementation did not affect (*P* ≥ 0.79) the reproductive outcomes: proportion of cows pregnant at 150 days postpartum, overall pregnancy per FTAI and overall pregnancy loss, and days for calving to conception interval recorded until 150 days postpartum (Table 5 and Fig 3).

**Table 5:** Effect of prepartum control or conjugated linoleic acids (CLA) supplementation on overall pregnancy per fixed timed artificial insemination (FTAI) and pregnancy loss, and proportion of cows pregnant at 150 days postpartum.

	Treatments^a^			*P-value*		
Item	Control	CLA	SEM	Trt^b^	Service	Trt x Service^c^
Pregnancy per FTAI, % (n_p_/n_s_)^d^	39.7 (96/240)	39.6 (106/263)	3.75	0.98	0.09	0.23
Pregnancy loss, % (n_l_/n_p_)^e^	11.7 (11/96)	13.1(13/106)	3.81	0.79	0.33	0.24
Cows pregnant at 150 days^f^, %	66.4 (85/128)	67.4 (93/138)	4.47	0.86	-	-

^a^ Treatments: Control (n = 128) cows supplemented with 78 g of Energy Booster 100 (Milk Specialties Global, Eden Prairie, MN) and CLA (n = 138) cows supplemented with 100 g/day of Lutrell Pure (BASF, Ludwigshafen, Germany). Supplementation was by individual topdress of product mixed with 200 g corn gluten feed for 16 days prepartum.

^b^ Treatment.

^c^ Interaction treatment x service.

^d^ Number of cows pregnant (n_p_) divided by total number of services (n_s_) until 150 DIM.

^e^ Number of pregnancy losses (n_l_) divided by total number of pregnant cows (n_p_) until 150 DIM. Pregnancy loss was calculated from 32 to 46 days after FTAI.

^f^ Proportion of cows pregnant at 150 days postpartum.

## Discussion

To our knowledge, no other published study has evaluated the effects of supplementing CLA isomers exclusively in the prepartum period in dairy cows. We hypothesized that CLA supplementation in the prepartum transition period would decrease fat mobilization around calving and improve metabolic health and lactation performance without reducing milk fat synthesis. This hypothesis was based on findings from a previous study (three treatments: no supplement, CLA supplemented pre- and post-partum, CLA supplemented postpartum only) which suggested there may be a possible effect of prepartum CLA supplementation on reducing lipolysis from occurring in adipose tissue [8]. In their study, CLA supplementation during both the pre- and postpartum period reduced plasma FA during the early postpartum period, compared to control cows or cows only supplemented postpartum. The authors attributed the lower serum FA to a lower BCS loss, which was also observed for pre- and postpartum CLA supplemented cows. While this experiment provided valuable insights, it did not have a treatment group with CLA supplementation only prepartum. Therefore, supplementing CLA exclusively during the prepartum period in the current study allowed the investigation of the effect of CLA on reducing blood FA in the peripartum period. Furthermore, the use of this strategy would not likely decrease milk fat synthesis since the half-life of plasma trans-10, cis-12 CLA and the half-life of enzymes responsible for milk fat synthesis subject to regulation by this CLA isomer are less than 3 days [9–11]. To examine the effect of treatment on animal productivity, health, and reproduction, we individually top-dressed the treatments to individual cows that were being fed a typical TMR on a privately-owned dairy. Similar methodology has been utilized in previous studies with daily top-dressing of rumen-protected choline [19] and methionine [20] or daily feeding of conjugated linoleic acid in an automatic feeding system in the milking parlor [21]. This method allowed individual feeding of CLA and maintenance of cow as the experimental unit and thus, increased statistical power; although DMI in individual cows could not be measured or regulated using this approach.

Prepartum supplementation with CLA was successful in achieving higher serum concentration of CLA isomers during the prepartum period. Daily CLA supplemented cows had a concentration of cis-9, trans-11 and trans-10, cis-12 CLA isomers 1.1 and 2.5 fold greater, respectively, compared to the control cows at 10 days prepartum.

As expected, cows supplemented with CLA did not experience milk fat depression. On the contrary, milk fat yield from calving to 60 days postpartum tended to increase in cows supplemented with CLA, which combined with the increased milk protein yield resulted in a higher milk energy output. A greater energy intake may have been the mechanism supporting the greater milk energy output in CLA supplemented cows.

Prepartum CLA supplementation did not affect BCS in the present study. Interestingly, serum FA was 30% and 15% lower at 1 and 7 DIM, respectively, for CLA supplemented cows compared to the control group. Since a variation in BCS was not observed with CLA supplementation, the decreased serum FA in CLA cows may have resulted from decreased internal body fat depot mobilization. This pattern was observed in a previous study when BCS was also unchanged, but CLA supplemented cows lost 29% less mass in the retroperitoneal adipose depot during the postpartum period compared to control cows [22]. The retroperitoneal adipose depot was the most sensitive tissue to mobilization compared to other fat depots in their study, suggesting that the conventional body condition score system may not always be conclusive to evaluate body fat loss [22].

Prepartum CLA supplementation decreased overall postpartum serum BHB by 13.1%, which resulted in a decreased prevalence of HYK on day 14 from 23.5% to 3.5%, although there was no difference at day 1, 7, and 30 postpartum. Literature on blood BHB and HYK prevalence in response to pre- and postpartum CLA supplementation are inconsistent. One study found a decreased prevalence of HYK when cows were supplemented pre- and postpartum with CLA compared to cows only supplemented postpartum with the same doses of CLA or control [8]. This was the only trial that compared pre- and postpartum supplementation with postpartum supplementation of CLA [8]. Other trials either did not find an effect [23, 24] or found an increase in plasma BHB in response to pre- and postpartum supplementation with CLA [5]. The decreased blood BHB observed in the current study could be reflective of decreased serum FA in the first week postpartum, as a potential fate of FA in the liver is incomplete oxidation to BHB when hepatic complete oxidative capacity is exceeded [1, 2].

Improvements in fertility have been observed previously pre- and postpartum CLA supplementation [21, 25, 26]. The mechanism of CLA to influence fertility has been associated with improved energy status [26, 27]. It was not possible to evaluate the energy balance in cows during the current study and despite decreased serum FA and prevalence of HYK, no differences in fertility were observed. It is possible that positive impacts on reproductive performance may require both pre- and postpartum CLA supplementation, or that the intensive Ovsynch protocol employed concealed any possible reproductive effects of CLA.

## Conclusion

Prepartum CLA supplementation tended to increase milk fat and increased milk protein yield, resulting in higher milk energy output during early lactation. Reductions in postpartum serum FA and BHB are suggestive of improved metabolic health. Together, these findings indicate that prepartum CLA supplementation may be a plausible strategy to positively influence postpartum performance.
